# Elucidating the Influence of Electrical Potentials on the Formation of Charged Oligopeptide Self‐Assembled Monolayers on Gold

**DOI:** 10.1002/cphc.202000988

**Published:** 2021-03-05

**Authors:** Joshua S. Gibson, Paula M. Mendes

**Affiliations:** ^1^ School of Chemical Engineering University of Birmingham Edgbaston Birmingham B15 2TT UK; ^2^ School of Chemical Engineering University of Birmingham Edgbaston Birmingham B15 2TT UK

**Keywords:** oligopeptides, self-assembled monolayer formation, potential-assisted assembly, X-ray photoelectron spectroscopy, electrochemical surface plasmon resonance

## Abstract

Self‐assembled monolayers (SAMs) based on oligopeptides have garnered immense interest for a wide variety of innovative biomedical and electronic applications. However, to exploit their full potential, it is necessary to understand and control the surface chemistry of oligopeptides. Herein, we report on how different electrical potentials affect the adsorption kinetics, stability and surface coverage of charged oligopeptide SAMs on gold surfaces. Kinetic analysis using electrochemical surface plasmon resonance (e‐SPR) reveals a slower oligopeptide adsorption rate at more positive or negative electrical potentials. Additional analysis of the potential‐assisted formed SAMs by X‐ray photoelectron spectroscopy demonstrates that an applied electrical potential has minimal effect on the packing density. These findings not only reveal that charged oligopeptides exhibit a distinct potential‐assisted assembly behaviour but that an electrical potential offers another degree of freedom in controlling their adsorption rate.

## Graphical abstract

**Self‐assembled monolayers** based on oligopeptides are promising materials for biomedical and electronic applications. Herein, studies on potential‐assisted assembly of charged oligopeptides are presented that reveal slower adsorption kinetics in comparison with those self‐assembled at the open circuit potential, without it affecting the packing density. These findings expand the parameter space available to control oligopeptide assembly.

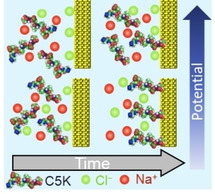

## 1 Introduction

Functionalised surfaces which have properties that respond to external stimuli have shown promise as the basis for novel applications of biological relevance including molecular[^1^, ^2^, ^3^] or DNA^[4]^ sensors, cell adhesion^[5]^ and DNA based molecular switches.^[6]^ Such surfaces have been termed “switchable surfaces”, due to the possibility of switching the surface properties, and have been developed to respond to a range of stimuli.^[7]^ Electrical potential,[^1^, ^2^] temperature^[8]^ and pH^[9]^ have all been demonstrated as methods of controlling the surface structure of self‐assembled monolayers (SAMs) temporally. As well as being able to control the behaviour of surface‐bound species in this way, it is also desirable to utilise similar interactions during surface formation allowing a greater control over the final surface structure.[^10^, ^11^]

A few studies have been performed into the effects of applying electrical potentials to the gold surface during SAM formation. When looking at the adsorption of uncharged thiol molecules from organic (usually ethanolic) solution it has been found that the application of a positive potential can increase the molecular packing density, hence generating a better quality SAM.[^12^, ^13^] This argument is supported by the notion that adsorption of thiols progresses through a one electron reaction, in which the sulphur donates an electron to the surface adsorbed gold atom, with a positive potential applied to the gold assisting this process.^[13]^ Recent work, however, has shown that application of either a positive or negative potential did not appear to improve the packing density of a thiol with a terminating ferrocenyl group.^[14]^ Care, however, must be taken when attempting to draw global conclusions on SAM formation based upon formation studies within different solutions, as the solvent polarity and the organosulphur solubility affect the SAM quality.^[14]^ An STM study into the adsorption of L‐cysteine in acidified aqueous media also found that higher quality SAMs were formed at positive potentials, compared to negative potentials,^[15]^ with a different study finding that negative applied potentials of different magnitudes had no effect on the adsorption kinetics of cysteine.^[16]^ The differing conclusions show that this reasonably simple interaction is still poorly understood, with better understanding vital to the development of self‐assembled chemical sensors.

**Figure 1:**
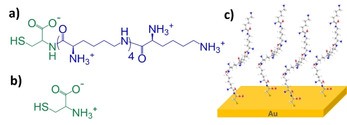
Chemical structures of the molecules used in this study: a) Cys‐Lys‐(ϵ‐Lys)_4_ (C5K) and b) cysteine (Cys). c) Schematic model of the C5K SAM based on a 3D C5K structure obtained by Chem3D.

Given that different organo‐sulphur molecules appear to have different adsorption behaviours on a gold surface, the aim of this work is to investigate the effect that the potential of a surface has on the kinetics of SAM formation, packing and structural stability of charged oligopeptides. The peptide C5K is chosen as the model charged oligopeptide since it contains five lysine groups, which impart the oligopeptide with a net positive charge, and a terminating cysteine residue for binding to gold (Figure 1). Such oligopeptides are of great relevance in the field of switchable biological surfaces, with studies on their adsorption properties being essential in the design of surfaces with tunable biospecific interactions.^[17]^ Following SAM formation under a passive incubation process, this type of oligopeptides have been previously shown to undergo conformational changes between collapsed and fully extended oligopeptide structures under an applied electrical potential.[^2^, ^3^] In the present study, we addressed the question, whether an electrical potential during oligopeptide SAM formation can influence the adsorption properties of this class of novel, charged oligopeptides. This new understanding of oligopeptide self‐assembly on surfaces has important implications to guide the design of materials and devices with switchable surfaces for use in several fields such as sensing, bioseparation, bioelectrocatalysis, biomedical research and delivery systems. In order to shed light on the characteristics of oligopeptide adsorption and how potential‐assisted deposition affect the oligopeptide chemisorption process, real‐time monitoring of the oligopeptide SAM adsorption kinetics onto the gold surfaces was performed using an electrochemical surface plasmon resonance (e‐SPR) setup. e‐SPR analysis were also complemented by X‐ray photoelectron spectroscopy and cyclic voltammetry to ascertain the chemical structure and surface coverage of the formed monolayers and their packing and structural stability.

## 2 Results and Discussion

**Figure 2:**
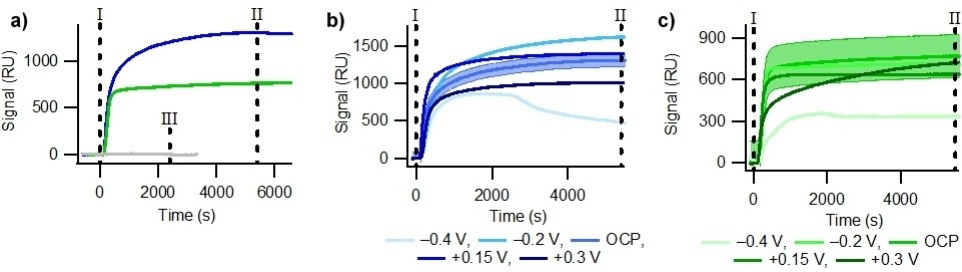
a) A comparison of the signal response during an e‐SPR experiment at the OCP for C5K (blue, solid), Cys (green, solid) and 1×PBS (grey, solid). Marker I indicates the beginning of the injection, while marker II indicates the end of the thiol injection and the flow cell being flushed with 1×PBS. Marker III shows the end of the 1×PBS blank injection. The e‐SPR responses for b) 0.1 mM C5K and c) 0.1 mM Cys injected when a gold surface is held at different potentials versus an Ag/AgCl reference electrode. Shaded areas represent the errors in repeated measurements for the OCP datasets, and can be taken as representative of all other potentials, with the calculated errors for each data set shown in Table 1. For all experiments the pH was 7.4 and the flow rate was kept constant at 20 μl min^−1^.

The experimental method for the e‐SPR studies involved an injection of either the running buffer or a thiol containing solution into the flow cell whilst a constant potential was applied, with Figure 2a showing the obtained signal responses as a function of time for experiments performed at the open circuit potential (OCP). For a ‘blank’ injection (grey solid line in Figure 2), where only the 1×PBS buffer is injected over the bare gold surface, only small deviations in the signal baseline (±10 RU) are observed. This confirms that there are not any new species being adsorbed onto the surface, with the noise representative or the error in the SPR setup. The measured OCP for all ‘blank’ injections at OCP on the piranha cleaned gold surfaces were within 60±20 mV, with randomly distributed fluctuations. Consequently there is no evidence any significant electrochemical change at the gold‐liquid interface during 1×PBS ‘blank’ injections.

For the injection of 0.1 mM C5K onto the bare gold under OCP conditions (Figure 2a, solid blue line) there is a significant increase (≈1300 RU) in the e‐SPR signal as a function of time, indicating a change in the near‐surface refractive index. A short time delay (≈200 s) exists between the start of the injection (marker I in Figure 2) and the e‐SPR signal increase, corresponding to the time required for the C5K solution to flow through the tubing between the injection loop and analysis flow cell. Upon the injection finishing (marker II in Figure 2) the e‐SPR signal decreases (≈15 RU) as the 0.1 mM C5K solution in the flow cell is replaced by 1×PBS running buffer causing a slight decrease in the solution refractive index. Removing the C5K solution from above the surface causes a decrease which is approximately 100 times smaller than the increase due to the C5K injection.

Adsorption experiments were also performed with 0.1 mM L‐cysteine (Cys) as a control molecule to compare with the positively charged C5K under the same adsorption conditions. Figure 2a shows that the C5K (blue solid line) induces a larger response, and hence a larger change in the near‐surface refractive index, than the Cys (solid green line) upon thiol injection, implying that there is a greater mass of C5K adsorbed than Cys. As the C5K (762 g mol^−1^) is a much larger molecule than the Cys (121 g mol^−1^), it is possible for a larger mass of C5K to be adsorbed onto the gold surface with fewer molecules adsorbed than in the Cys monolayer. The rate of increase for the SPR signal for the Cys adsorption is seen to be higher than that of the C5K (taking 140 s and 210 s to reach 50 % of the maximum coverage, respectively), suggesting that the adsorption kinetics are faster. Given that there is not a sharp decrease after the thiol injection has finished, and the surface is flushed with 1×PBS, it is concluded that both of the thiols are bound to the surface, rather than simply physisorbed. This bond is likely to exist as the formation of a gold‐sulphur bond between the polycrystalline gold surface and the cysteine terminal group, and is later supported by electrochemical studies.

**Figure 3:**
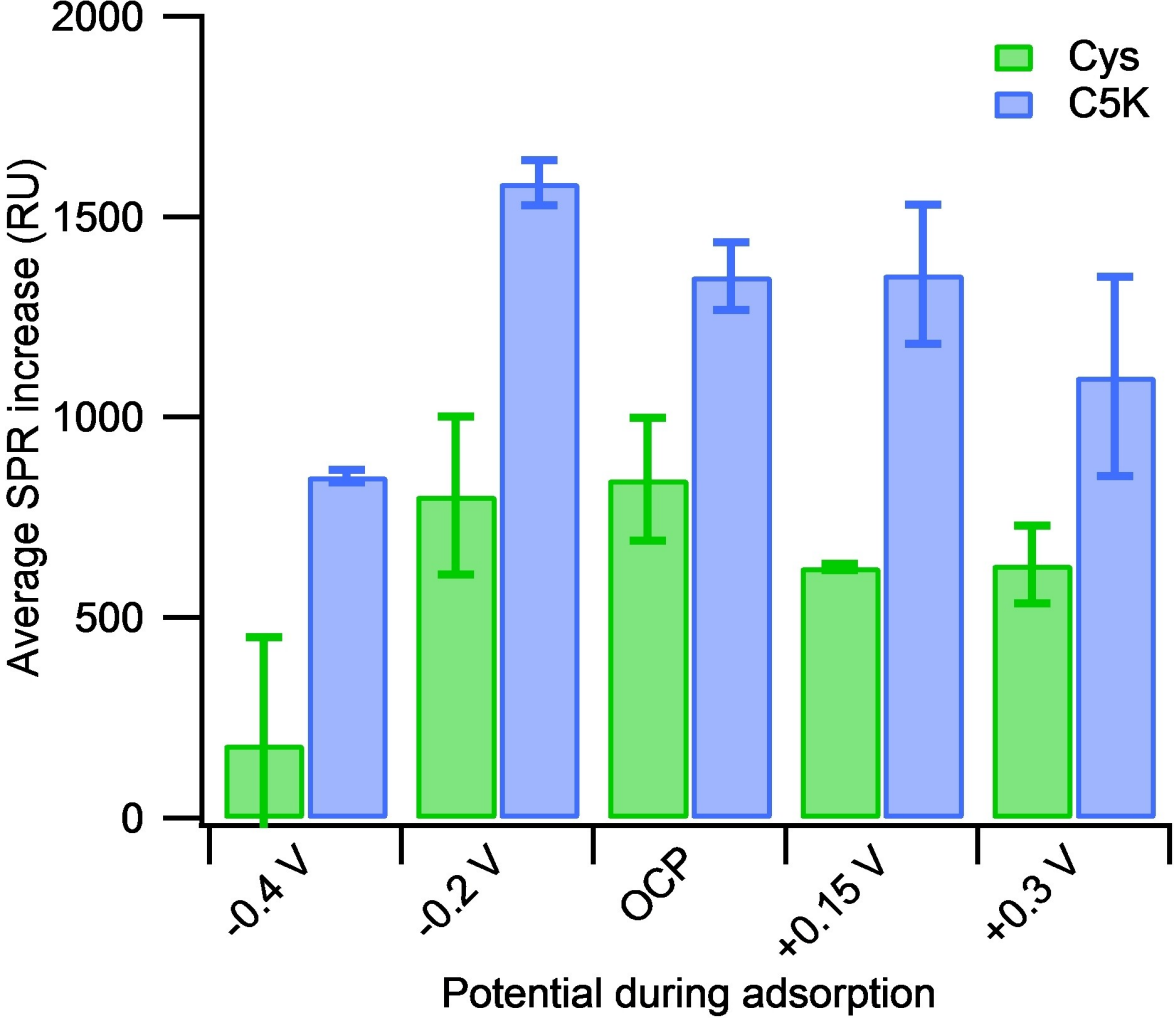
A comparison of the average signal increase during e‐SPR experiments for the different applied potentials when injecting 0.1 mM Cys (green, left) and C5K (blue, right). Averages have been calculated from at least two measurements, with errors equal to two standard deviations. The changes in the SPR signal were measured inside the window of the applied potential.

Injections of 0.1 mM C5K and Cys onto bare gold surfaces were performed whilst applying potentials of −0.4 V, −0.2 V, +0.15 V and +0.3 V vs Ag/AgCl. A plot of the signal responses for injections at these potentials is shown in Figures 2b and 2c for the C5K and Cys, respectively. It can be observed that applying different potentials to the surface appears to have a more significant effect on the total SPR change for C5K than for the Cys adsorption, however all changes are still within the experimental error (2 standard deviations) of repeated sensorgrams (Table 1 and Figure 3). This is highly likely to be due to the fact that polycrystalline gold substrates are being used, where it has been reported that adsorption under exactly the same conditions can lead to differing surface coverages.[^13^, ^18^] To reduce the effects of polycrystalinity on the SPR results studies on gold single crystals grown on glass would need to be performed.

**Table 1:** Signal changes for the e‐SPR responses observed under different applied potentials (−0.4 V, −0.2 V, OCP, +0.15 V and +0.3 V). Values have been calculated by taking the difference in the average signal between two times; the first after the injection has finished and the second before the injection had occurred, as discussed in the main text. Errors in the average value have been calculated as twice the standard deviation of the mean (2σ).

Applied potential	Average signal change and error [RU] for C5K	Average signal change and error [RU] for Cys
−0.4 V	562±225	185±267
−0.2 V	1580±65	806±197
OCP	1348±87	846±154
+0.15 V	1357±174	627±8
+0.3 V	1102±248	633±97

The differences in the magnitude of the SPR increase can be used to estimate the molecular footprints of the two thiols. Using the relationship^[3]^ of 1000 RU being approximately equal to 1 ng/mm^2^ of material and using Figures 2b and 2c to estimate signal increases of 1200 RU and 700 RU for C5K and Cys adsorption, respectively, the area per molecule can be found to be 1.06 nm^2^ for C5K and 0.287 nm^2^ for Cys. The footprint found for Cys agrees well with previous findings for thiol areas of 0.214 nm^2.[19]^ A larger footprint for C5K is to be expected given the length of the tail and the subsequent increase in the free volume required for motion of this tail. Additionally, under the pH 7.4 conditions used here, the lysine tail has a net positive charge, which may cause a further increase in the free volume due to electrostatic repulsions by neighbouring C5K. Aside from the differences in the magnitude of the SPR increases for C5K and Cys injections, the data shown in Figures 2b and 2c at each of the applied potentials all have similar shapes.

Similarly, their corresponding ‘blank’ injections (Figure S1 in the Supporting Information) also have appearances comparable to that shown in Figure 2a for the OCP conditions, confirming a lack of molecular adsorption in the absence of the thiol (C5K or Cys). The average increase in the SPR signal for thiol adsorption under the application of different potentials is shown in Table 1 and Figure 3, where it can be seen that the differences between the responses at different potentials are within the errors (shaded regions in Figure 2) found from the repetitions. The change in the SPR signal is larger for all C5K adsorption data than for the Cys adsorptions, apart from the −0.4 V datasets, which will be discussed later. When the errors are considered, it is found that there is no statistically significant difference in the total SPR response for either the C5K or the Cys when the potential used for adsorption is varied between −0.2 V and +0.3 V vs. Ag/AgCl. This result is surprising as it suggests that there is no influence of the applied potential on either the C5K, with a net charge, or the uncharged Cys. This invariance is in contradiction to some of the literature, which suggest that application of a positive potential leads to a more packed monolayer surface for uncharged thiols with faster adsorption kinetics,[^12^, ^20^, ^21^] but agrees with literature stating that application of a negative potential does not affect cysteine adsorption.^[16]^

Studies into the kinetics of adsorption, typically looking at lower thiol concentrations, show that when using 20 μM thiol the surface coverage reaches 30 % in 0.5 s while at 1 mM the surface coverage reaches 60 % in 0.5 s and 95 % in 1 s.[^22^, ^23^] For the 0.1 mM concentration used here, which is comparable to the latter aforementioned study, it is likely that the thiol surface reaches its maximum coverage within a few seconds. Hence the potential does not have a significant length of time to affect local thiol concentration through molecular migration and the resulting adsorption process. This finding is supported by other work, which has found that the application of a positive potential (+0.1 V to +0.8 V vs. SCE in ethanol) only affects the adsorption of an uncharged thiol, at 1 mM, for the first 0.2 seconds, when monitored by chronoamperometry.^[21]^ It is, however, possible that the applied potential affects the molecular orientation or the strength of the Au−S bond, possibly altering energy barriers to surface migration or reorientation.

The use of an SPR flow cell in this work, rather than a near‐instant addition of thiol to a static cell, is believed to contribute to the slower monolayer formation seen here than in literature (which plateaus after ≈10 seconds). Since thiol is introduced slowly (at 20 μl min^−1^ in a cell with a volume ≈100 μl) a gradual rise for the SPR signal can be expected, rather than an instant increase. For experiments where adsorption occurs under potential control the surface was charged 10 minutes prior to the injection of the thiols, to prevent the set‐up of an electrical double layer interfering with the signal change due to adsorption, see Figure S1. It is proposed that the electrical double layer will have the same structure for the bare gold electrode and the C5K or Cys functionalised gold electrodes, for a fixed applied potential. Since the concentration of ions in the 1×PBS supporting electrolyte is about 1500 times that of the C5K molecule it is reasonable to assume that the electrical double layer is formed solely of PBS ions and the addition of C5K or Cys does not affect the ion concentration significantly enough to affect the layer structure. For both thiols studied here the adsorption at +0.3 V (darkest lines in Figures 2b and 2c) and −0.4 V (lightest lines in Figures 2b and 2c) appear to have a less steep increase, and hence slower rates of adsorption than the other, less extreme, potentials. It is possible that this is due to the presence of an electrical double layer which is set up at the electrode interface, consisting of the ions within PBS.

As the magnitude of the potential increases, so too does the packing of the electrical double layer, therefore providing more resistance to thiol adsorption.^[24]^ The applied potential, however, appears to affect the kinetics of adsorption for the C5K, but not the final monolayer density. Only the adsorption kinetics at +0.3 V and −0.4 V are observed to be different for the Cys, while all potentials are affected with C5K, suggesting that the effect is not solely due to the setup of a thick electrical double layer. Such an effect rises from the charge on the molecule supporting the idea that migration of the charged C5K ions under the applied potential can drive the kinetics of adsorption. Such kinetic control, through an applied potential, may be applied in multi‐component systems, especially where one component is charged, or can be made so by pH changes. In such a case the applied potential may affect the surface coverage ratio of the different components.

It is evident that there is a high level of complexity within these thiol‐buffer systems, and that the role of the supporting electrolyte can have on the SAM formation cannot be ignored. The importance of the role that buffer ions play in the adsorption of thiols has been seen previously. Increased clustering in DNA SAMs was observed to occur more readilly in the absense of chloride anions,^[25]^ whilst the use of pulsed potentials during both single strand DNA and alkanethiol SAM formation was observed to promote fast ion stirring, and rapid formation of dense layers.[^26^, ^27^] In addition, the optimal conditions for the SAM formation (potential pulse amplitude and duration) were seen to vary with the alkanethiol length and would be expected to vary with the supporting electrolyte used.

**Figure 4:**
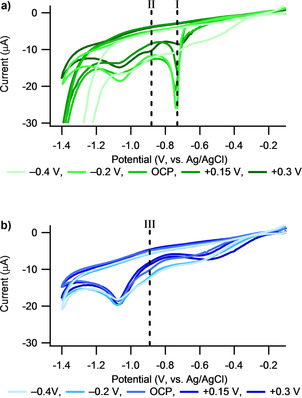
A comparison of desorption CVs collected from the a) Cys and b) C5K surfaces after a 90 minute incubation in 0.1 mM thiol solutions made in 1×PBS. CVs were collected in 0.1 M KOH at a scan rate of 100 mV s^−1^ vs. Ag/AgCl. The reduction processes denoted by I, II and III are attributed to thiol desorption processes, as described in the text.

A similar effect, in which the rate of adsorption appears reduced, can also be seen for −0.4 V, however the shape of the SPR plots are also different for this potential. The decrease in the SPR signal observed after layer formation at −0.4 V for both the Cys and the C5K surface is believed to be due to electrochemically induced desorption of the thiols from the gold surface. There is initial adsorption of the thiols to the gold surface, as indicated by the initial increases in the SPR datasets in Figure 2, prior to a decrease after a critical surface concentration is reached. This hypothesis is supported by cyclic voltammetry results, Figure 4, of the SAMs, collected in 0.1 M KOH vs. Ag/AgCl, after their formation in the e‐SPR cell.

Figure 4a shows the CVs for the Cys SAMs, with the sharp peak (I) at ≈−0.7 V corresponding to reductive desorption of cysteine from the gold surface.[^28^, ^29^] For all CVs collected from the Cys SAMs formed at −0.4 V, peak I is either greatly reduced in size or missing completely, with no other peaks observed that have been ascribed to the desorption process. The lack of a significant cysteine desorption peak for surfaces formed at −0.4 V implies that there is not a large enough quantity of Cys still adsorbed to the gold surface to provide a significant response. For adsorptions performed with Cys at +0.3 V a reduction in the area of peak I is seen, and a new peak arises at −0.88 V (peak II). The position of this new peak is thought to be due to adsorption of Cys in a more stable surface state, promoted by the larger applied potential reducing the potential energy landscape for adsorption. The presence of an electrochemical desorption peak due to the reduction of sulphur is further evidence of the formation of a gold‐sulphur bond between the Cys/C5K and the gold surface.

It is difficult to observe the reduction in size of the electrochemical desorption peak for C5K at −0.89 V (denoted by III in Figure 4b) as the peak is about ten times smaller than for Cys and is not as well resolved. Under the assumption that the thiol desorption mechanism is the same, with literature suggesting desorption occurs through a one electron reduction,[^18^, ^30^] the C5K packing density is observed to be ten times lower than that for Cys. This estimation is slightly different to the footprint calculation based off of the SPR response which suggests that the C5K has a footrpint that is four times that of the Cys. These observations are consistent with the fact that the C5K will have a larger free volume than the Cys due to the motion of the lysine‐containing tail, meaning that the Cys would be expected to pack more closely on the gold surface than the C5K can.

In addition to the increased free volume, the C5K tail also has a positive charge at pH 7.4 which would be expected to provide lateral repulsions between neighbouring C5K molecules, reducing the packing density. Conversely, Cys molecules have been seen to pack more tightly due to the attractive lateral interactions that arise from the alignment of the negatively charged carboxylate groups and the protonated amines.^[16]^ It may be expected that the applied potential could affect the conformation of the C5K molecule, such that it would be more upright for positive surface potentials and lie down for negative potentials, as seen in previous works.[^2^, ^3^] This may be expected to lead to an increase in the surface coverage, as measured by the electrochemical desorption peak or the SPR signal increase, but no such evidence was found. It is possible that the large relative charge (up to+5*e*) on the C5K acts to oppose too high a packing density due to the electrostatic repulsion, consequently having a significant effect on structure of the adsorbed layer. The natural differences in the adsorption coverages due to the use of polycrystalline gold surfaces, as seen previously[^13^, ^18^, ^31^] may make observing such subtle changes difficult.

**Figure 5:**
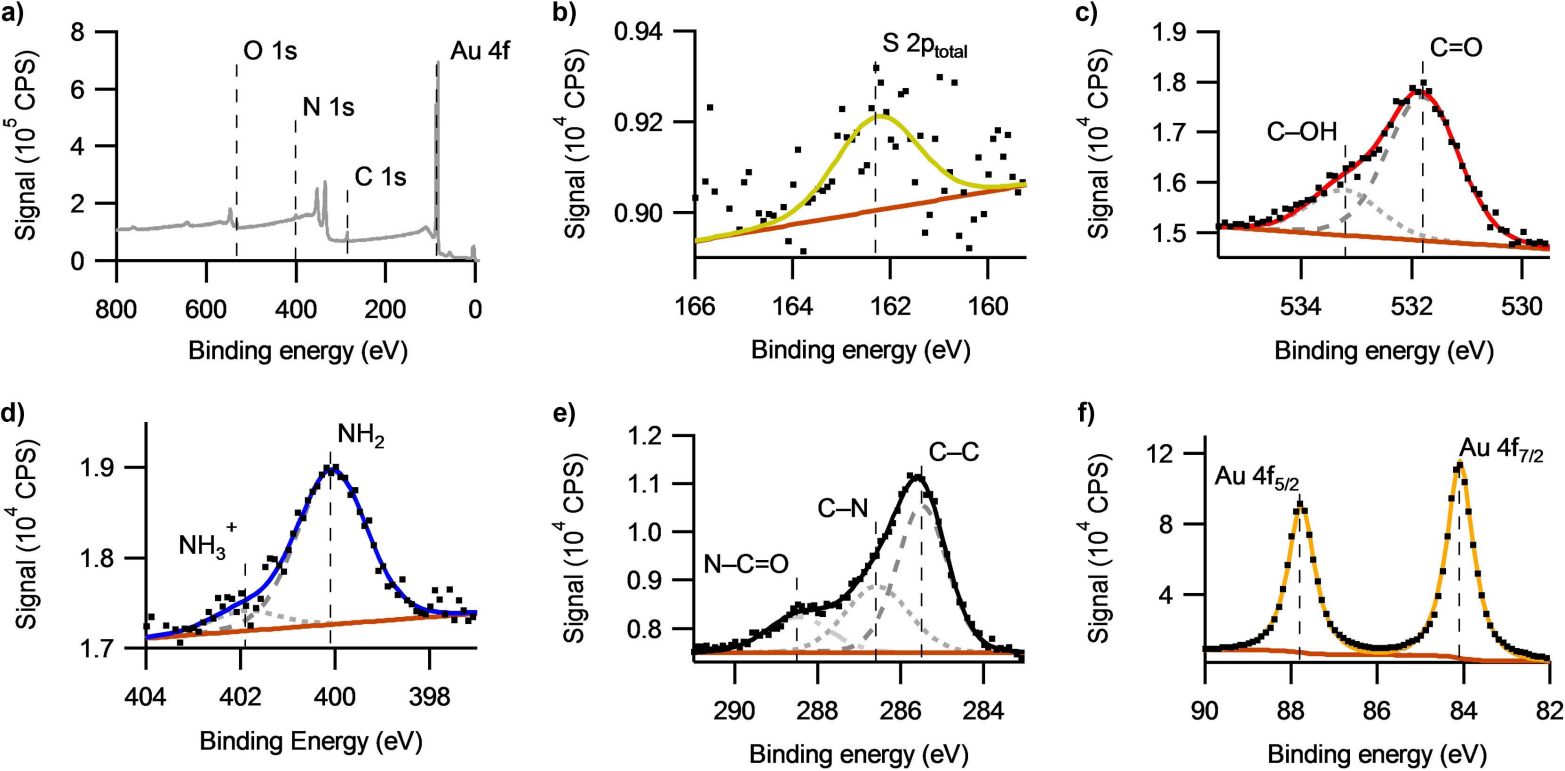
Electron distribution curves for a C5K surface formed within the electrochemical cell after a 90 minute incubation at the OCP, versus a Ag/AgCl reference electrode. The survey scan (a) shows all the expected peaks, except the S 2p due to the low intensity (Au 4d (≈340 eV) and 4p (≈550 eV) doublets are not labelled). The (b) S 2p, (c) O 1s, (d) N 1s, (e) C 1s and (f)Au 4f EDCs are each fitted with a background (brown line) and components (grey dashed/dotted lines), with the sum of the fitted components shown with the coloured line.

To compliment e‐SPR studies, C5K surfaces formed under an applied potential were studied by X‐ray photoelectron spectroscopy (XPS), with the collected electron distribution curves (EDCs) for the OCP incubation shown in Figure 5. The EDCs collected for all other potentials are similar to those shown in Figure 5. Survey spectra do not show any unexpected elements, with the high‐resolution EDCs having the shapes and number of components expected for the C5K, based on the stoichiometric ratios (SC_33_N_11_O_7_H_67_). The presence of the S 2p peak at 162.2 eV is consistent with sulphur from an environment bound to gold as confirmed by other studies on cysteine.^[32]^ Evidence of a small quantity of carbonaceous contamination exists within the C 1s and O 1s regions. Fitting constraints in the relative binding energies and full‐width at half‐maximum were used for the high resolution EDCs to ensure that the same model was used across all studied samples, however all of the intensities were allowed to vary, with the exception of the spin‐orbit split doublets, see Table S1. The signal in the S 2p region is quite low, due to the low surface concentration, so the S 2p doublet cannot be resolved and is fitted with a single peak, with the lack of a peak corresponding to ‘unbound’ sulphur indicating the absence of multilayers.

**Figure 6:**
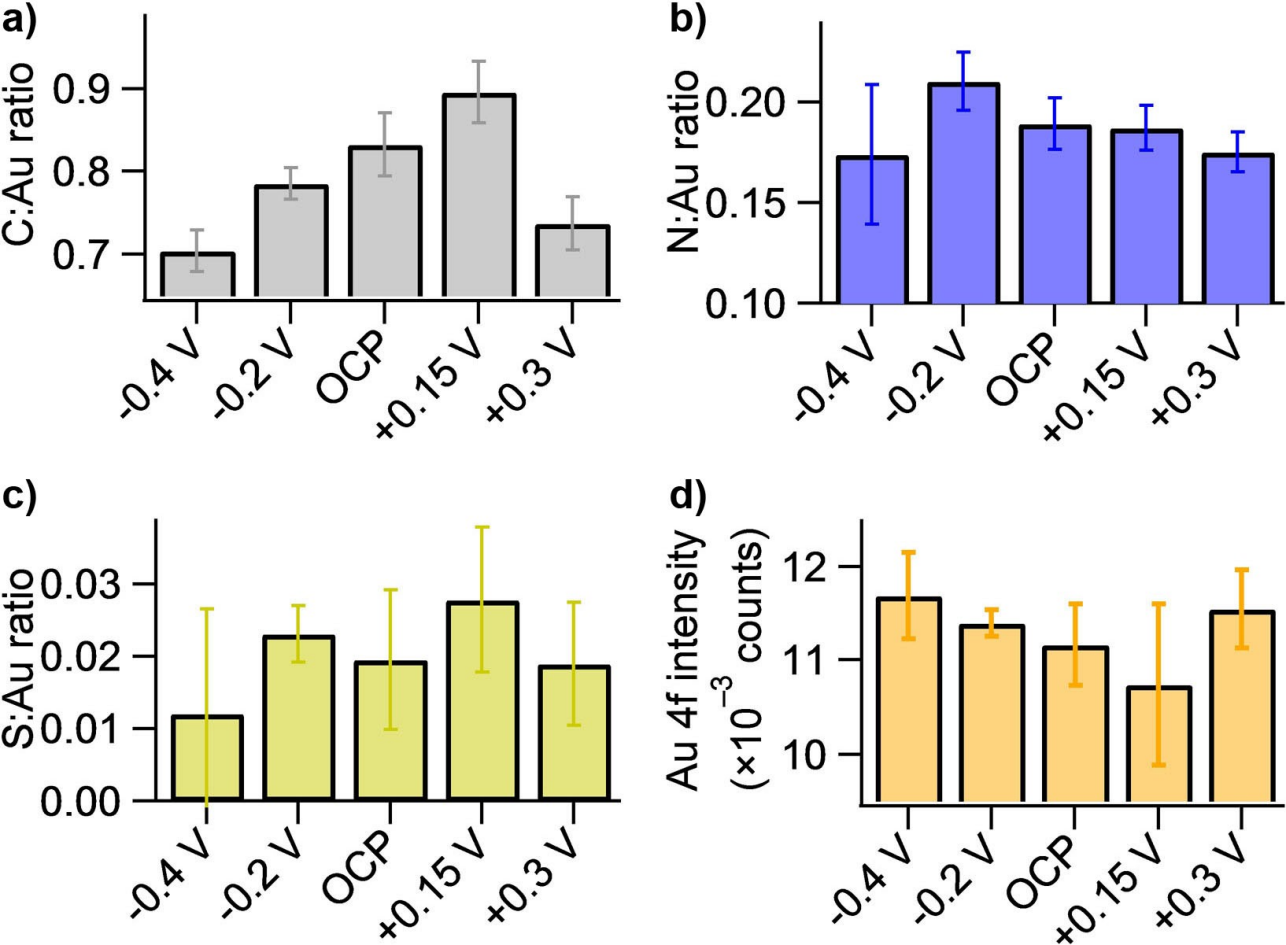
Plots showing the ratio of C5K photoelectron peaks to the Au substrate peaks for C5K surfaces formed at different potentials, versus the Ag/AgCl reference electrode, during the 90 minute incubation. Ratios are shown for (a) C 1s : Au 4f, (b) N 1s : Au 4f, (c) S 2p : Au 4f and (d) showing the Au 4f substrate signal. The data is an average of three measurements, with the error being equivalent to two standard deviations (2σ).

The peak areas from the XPS fitting were used to assess the quantity of C5K adsorbed to the gold surfaces under differing adsorption conditions, and compared to the e‐SPR results. Ratios of the C5K photoelectron peak areas to the gold substrate, shown graphically in Figure 6a–c, were used to estimate the relative surface coverages obtained at different potentials. The gold intensities, Figure 6d, suggest that there is the least amount of material on the −0.4 V surface, however due to the small number of adsorbed species the experimental error (±500 counts) is comparable to the observed differences (±500 counts) at different potentials. The ratios of the total C 1s photoelectron peak to gold (Figure 6a) shows a different pattern to the ratios of nitrogen and sulphur, indicating that there may be some additional carbonaceous species on the surface. It is well known that the carbon and oxygen signals are the most susceptible to adventitiously adsorbed contamination, due to atmospheric carbon dioxide and hydrocarbons. For the data collected here the intensity of the C−C environment (which can be used to estimate carbonaceous contamination, see Figure S2) is highest, while the C−N and N−C=O environments do not show as much variation between samples, which is consistent with adventitious adsorption variations between the samples. It is observed that there is also an invariance of the N 1s: Au 4f and S 2p: Au 4f ratios for the potential range of −0.2 V to +0.3 V, with all the values being within error of each other. This result suggests that the only difference between samples may be the amount of adventitious carbonaceous adsorption, not the variation in the amount of adsorbed C5K.

Furthermore, this supports the findings from the e‐SPR, that there is not any significant effect of the applied potential on the total amount of C5K that is adsorbed. Table 2 shows the ratios of the N 1s peak to the Au 4f and the S 2p to the N 1s peak intensities. It can be seen that the N 1s/Au 4f ratios are similar, and within error of each other and the value of 0.19. The ratio of S 2p/N 1s is similar to the theoretical value (based on the stoichiometry) of S/N (1/11=0.09), for all of the potentials. When −0.4 V is applied to the surface during adsorption it appears that there is a slight depletion of sulphur, in comparison to the nitrogen and a slight depletion of nitrogen when compared to the gold, if we ignore the larger errors for these values. The large errors are thought to arise from the inconsistent desorption of the C5K from the polycrystalline gold surface, with different surface domains having slightly different potentials for thiol desorption.[^28^, ^30^]

**Table 2:** Ratios of the integrated N 1s XPS peaks to the Au 4f and S 2p peaks to the N 1s peak areas for the surfaces formed at different applied potentials (−0.4 V, −0.2 V, OCP, +0.15 V and +0.3 V). A larger N 1s/Au 4f ratio indicates that the amount of nitrogen, and hence bound C5K, is greater. Errors in the average value have been calculated as twice the standard deviation of the mean (2σ).

Applied potential	Ratio of XPS signals N 1 s/Au 4f	Ratio of XPS signals S 2p/N 1 s
−0.4 V	0.17±0.04	0.07±0.08
−0.2 V	0.21±0.02	0.11±0.02
OCP	0.19±0.01	0.10±0.05
+0.15 V	0.19±0.01	0.15±0.05
+0.3 V	0.18±0.01	0.11±0.05

Conclusions based on the electrochemical experiments alone must be interpreted with care as, due to the setup used with the e‐SPR flow cell and the use of polycrystalline substrates,[^13^, ^18^] the CVs can only be trusted as a qualitative tool. The reduced ratios of S/Au and S/N obtained in XPS for −0.4 V incubations, compared to the other potentials (Figure 6c, Table 2), also support the conclusion that there is electrochemical desorption of C5K from the gold surface at −0.4 V. A decrease in the value of the S/N ratio (if the error is not considered) may seem to imply that there is loss of sulphur, but not of nitrogen, suggesting some form of molecular fission event where the S group leaves and the N group remains. Given the unlikelihood of this type of molecular fission it could be reasonable to assume that this apparent reduction in sulphur intensity at −0.4 V is related to the low signal to noise ratio of the S 2p peak, hence leading to the larger error in the S/N ratio.

The effective thickness of the C5K layer on the gold surface can be calculated from consideration of the scattering of the emitted Au 4f photoelectrons by the adsorbed C5K overlayer, and the subsequent attenuation of the Au 4f signal. Using the Beer‐Lambert law for photoelectron attenuation at normal emission, shown in Equation (1), the effective layer thickness, *d*, can be found. The effective layer thickness is the thickness of a hypothetical layer of homogenous material that has the same photoelectron scattering properties as the real‐world layer, which itself may not be a uniform layer (but instead islands or patches).

(1) $$I_{d}=I_{0}exp\left(\frac{-d}{\lambda }\right)$$

**Figure 7:**
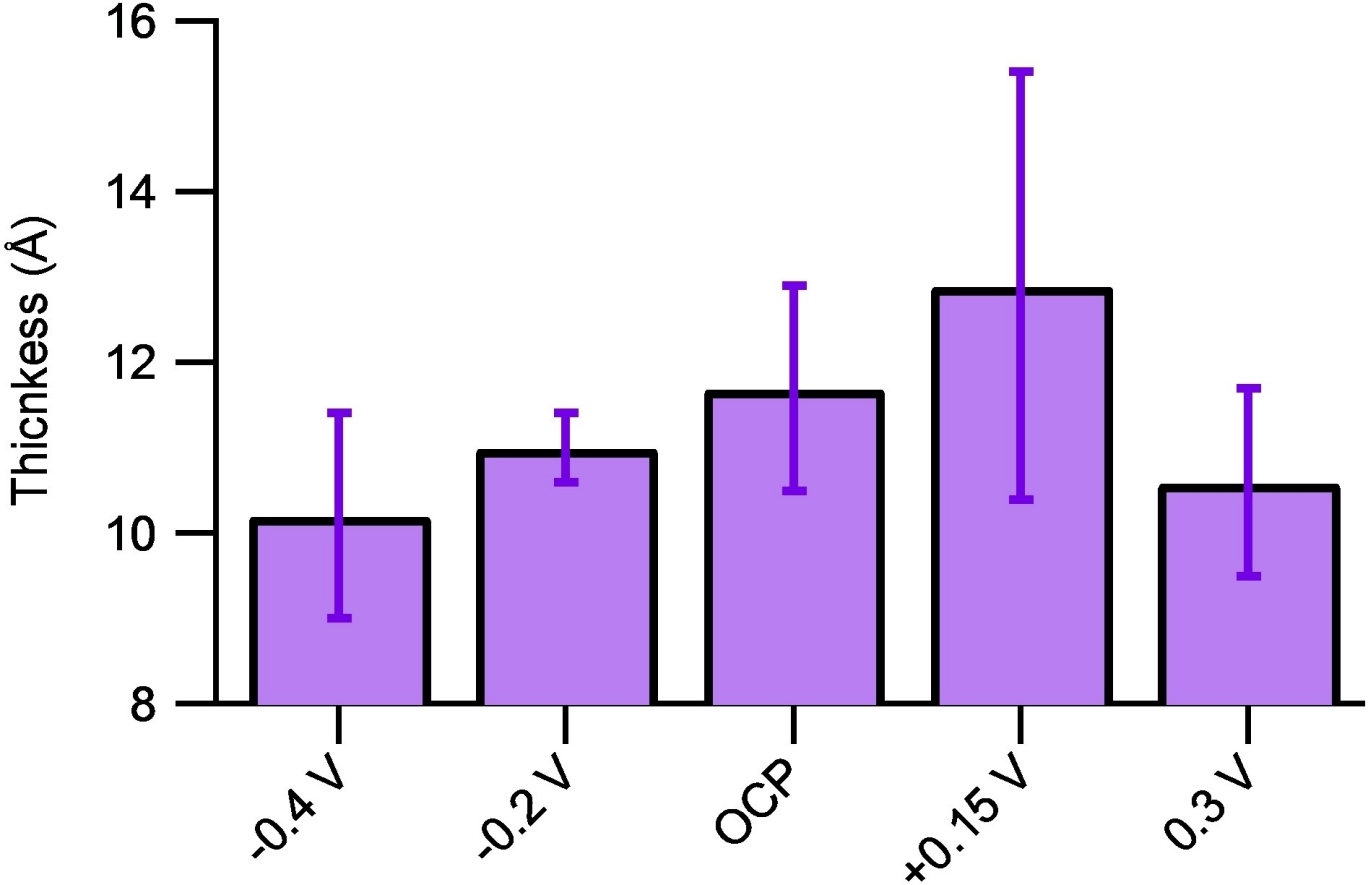
The average film thickness values calculated from the attenuation of the Au 4f signal by the surfaces formed at different potentials, versus the Ag/AgCl reference electrode, during the 90 minute C5K incubation. The attenuation length used was 32.04 Å. Values are the average of three measurements, with errors calculated as twice the standard deviation (2σ).

The attenuation length, *λ*, can be approximated as 0.8× the inelastic mean free path,^[33]^ which itself is found through the TPP predictive model.^[34]^ For Au 4f photoelectrons (kinetic energy of 1402.7 eV when an Al Kα source is used) travelling through C5K, the attenuation length was predicted to be 32.04 Å. The intensity of the bare gold, *I_0_*, is obtained from the Au 4f peak area of an uncovered gold surface (sputtered clean, see Figure S3), with minimal carbon contamination, while the intensity of the covered surface, *I_d_*, is measured for each of the surfaces formed under potential control. The measured effective film thicknesses are shown in Figure 7 and are shown to all be within error of each other, supporting the conclusion that the application of the potential to the gold surface during the injection of 0.1 mM C5K does not affect the amount of material that adsorbs.

It should be noted that these film thicknesses are affected by the amount of adventitiously adsorbed carbon on each of the C5K surfaces, leading to some variability in the effective film thickness. Interestingly the presence of the C5K appears to have prevented significant quantities of carbonaceous contamination on the surfaces, with the carbon contamination on the gold surface prior to sputtering being approximately ten times the carbonaceous contamination on the C5K surfaces (see Figure S3 for details).

The film thicknesses measured for the C5K using XPS are all within the range 11.5±1.5 Å, whilst the C5K is predicted to be about 50 Å long, if fully stretched, as determined by the ChemDraw software. Given that the e‐SPR shows that the signal had plateaued, meaning that the C5K surface had saturated, such that no more C5K can adsorb, these XPS results confirm that the lysine chains are not fully stretched. By treating the surface as having two surface domains, one covered by fully extended C5K chains (such that *d*=50 Å) and the other with no material adsorbed on it (such that the gold photoelectrons from that domain are not attenuated), a modified photoelectron attenuation equation, eq. (2), can be used to find the proportion of the surface covered by the fully extended C5K, *θ*.

(2) $$I_{d}=\left(1-\theta \right)I_{0}+\theta I_{0}exp\left(\frac{-d}{\lambda }\right)$$

Using this modified equation the C5K surface coverage is calculated as 0.381 meaning that 61.9 % of the gold is uncovered, assuming the C5K is fully extended. It is expected that in reality the C5K chains are folded somehow, so it is unlikely that there is any significant quantity of bare gold showing in solution as this would likely promote further adsorption or higher levels of surface contamination (see Figures S2–3). To find the degree of folding we utilise the assumption that C5K can be folded over in such a way that if the molecule length decreases by a factor, *n*, its footprint increases by that same factor (i. e. a molecule folded in half, has half the original length but twice the original footprint). It was found that for a C5K with quarter of the length, and a footprint that is four times larger then, by using a modified format of Equation (2) the coverage of C5K/sulphur groups can be found to be 0.233, with only 6.7 % of the gold uncovered. This simplistic methodology also seems to agree with the previous findings from the e‐SPR; the C5K has a footprint that is approximately four times that of the Cys. Our finding, that the C5K coverage is between 0.2 and 0.4, from the XPS data agrees with previous adsorption studies on cysteine which has also been found to have a coverage of 0.3±0.1 on Au(111), for a complete monolayer.^[32]^

## 3 Conclusions

Studies into the adsorption of charged oligopeptides onto a gold surface under potential control have found that the adsorption properties of these oligopeptides are not the same as for other, simpler, long chain alkanethiols. The application of an applied potential in the range −0.2 V to +0.3 V did not appear to affect the overall monolayer packing density for the C5K, whilst with alkanethiols small positive potentials have been seen to enhance the surface packing. Although the applied potential was not seen to affect the final monolayer packing density, it did appear to affect the adsorption kinetics in two different ways. At more extreme potentials the presence of a thicker electrical double layer restricts the diffusion of the thiols to the surface, and can be seen to affect both the C5K and Cys adsorption rates. In the case of the charged C5K molecules there is an additional contribution of the potential to the adsorption rate as the applied potential affects the orientation of the charged tail, restricting the ability of other molecules to adsorb nearby. Such behaviour allows for the possibility to control the concentrations of surface adsorbed species when looking at adsorption from multi‐component solutions, especially if one of the components can be charged (i. e. by a pH change). The adsorption rate of the charged molecule can be expected to depend on its molecular dimensions and charge density. Electrochemically stimulated desorption for both C5K and Cys was seen to occur at −0.4 V in the 1×PBS solutions after the initial adsorption of thiols to the surface, with studies at more negative potentials not expected to show any adsorption at all due to the instability of the Au−S bond. The film thickness and surface coverage of adsorbed C5K molecules have been evaluated by XPS measurements, accounting for the conformational changes of the long C5K molecules, and are seen to be consistent with e‐SPR measurements and previous studies of cysteine on gold surfaces. We believe that our findings on the potential‐assisted assembly of oligopeptides have important implications for the development of multicomponent, functional surface materials, wherein an electrical potential expand the **parameter** space available to **control** oligopeptide **assembly**.

## Experimental Section

### Chemicals

The oligopeptide Cys‐Lys‐(ϵ‐Lys)_4_ (C5K, Figure 1a) was purchased from Protein Peptide Research Ltd. (UK), and used with no further treatment. L‐cysteine (Cys, >98 %, ACROS organics, Figure 1b) was used as received. Phosphate buffered saline (PBS) 10×solution (Fisher Scientific International Ltd.) was diluted by a factor of 10 in Milli‐Q water (Merck Millipore Direct‐Q 3 UV) and had its pH adjusted to 7.4 (if necessary, using either aqueous HCl or NaOH solutions), before being passed through a 0.45 μm PVDF membrane filter (Durapore^®^) and degassed under vacuum. Aqueous 0.1 mol dm^−3^ KOH (analytical reagent grade, Fisher Scientific UK Ltd.) solution was made in Milli‐Q water, and purged with argon (99.999 %, British Oxygen Company) prior to use.

### Electrochemical Surface Plasmon Resonance (e‐SPR) Experiments

SPR experiments were performed using a Reichert Technologies system (SR7000 DC detector, SR7300 semi‐auto valve and SR7500 pump) with a dual channel cell with the channels in series. Plain gold on glass chips (Reichert Technologies) were cleaned in piranha solution for at least 7 minutes and rinsed thoroughly in water prior to being dried in an argon stream. Chips were then inserted into the system and allowed to stabilise in the running buffer for approximately 90 minutes prior to adsorption experiments. The system was using a specially designed electrochemical flow cell (Reichert Technologies), with the gold surface acting as the working electrode (0.12 cm^2^ per channel), a platinum wire counter electrode (≈0.071 cm^2^) and a silver‐silver chloride (BASi, 3 mol dm^−3^ NaCl) reference electrode. A Gamry Instruments Reference 600 potentiostat was used to control the potential of the gold chip, whilst simultaneously performing chronoamperometry experiments, and to measure the open circuit potential (OCP) during adsorption experiments. The running buffer used was filtered and degassed 1×PBS at pH 7.4, flowing at 20 μl min^−1^, whilst during desorption experiments argon purged 0.1 mol dm^−3^ KOH was injected into the flow cell. Desorption experiments were performed by sweeping the potential from −0.1 V to −1.4 V, vs. Ag/AgCl at 100 mV s^−1^, as an added measure to confirm the presence of adsorbed thiol to the surface.

### Peptide Adsorption Studies

Peptide (L‐cysteine and C5K) adsorption was studied as a function of time whilst different potentials were applied to the e‐SPR cell. Adsorption studies were performed at −0.4 V, −0.2 V, OCP, +0.15 V and +0.3 V vs Ag/AgCl. Experiments consisted of an initial dissociation stage on bare gold, to identify a baseline, followed by the application of the potential, during which an injection was performed. After the injection had finished the applied potential was turned off, and a final dissociation stage was performed. A constant flow rate of 20 μl min^−1^ was used for these experiments. Concentrations of 0.1 mM were used for the L‐cysteine and C5K injections, while 1×PBS injection stages were also performed to allow comparisons between peptide and ‘blank’ injections to be made.

### X‐ray Photoelectron Spectroscopy (XPS) Characterisation

Surfaces terminated with the C5K oligopeptide were also formed on gold on silicon substrates (100 nm Au on Si(100), Georg Albert PVD) within a conical electrochemical cell with an opening at the bottom, which sat on the gold surface to define the electrode area with an O‐ring (2.01 cm^2^). A platinum wire counter electrode (≈1.5 cm^2^) and a silver‐silver chloride (BASi, 3 mol dm^−3^ NaCl) reference electrode were also used in this cell. Surfaces were prepared in a similar way to the ones described in the SPR experiments. Initially the piranha cleaned (7 minutes) gold substrates were loaded into the electrochemical cell and submerged in 1×PBS and then a potential was applied. After this the C5K was added to the electrochemical cell to a concentration of 0.1 mM and the incubation was performed. After 90 minutes the cell was flushed out with 1×PBS, whilst still under potential, to remove any unbound C5K after which the chip was rinsed in milli‐Q water and then dried in argon. C5K surfaces were formed at potentials of −0.4 V, −0.2 V, OCP, +0.15 V and +0.3 V vs Ag/AgCl. Samples were loaded into a Thermo NEXSA with an Al Kα photon source (1486.7 eV, 19.2 W) with analysis being performed below 5×10^−8^ mbar. The gold on silicon samples were attached to the substrate baseplate with copper clips to prevent surface charging distorting the electron distribution curves (EDCs). Survey scans (pass energy of 150 eV, step size of 0.5 eV) and high resolution scans (Au 4f, S 2p, C 1s, N 1s, O 1s, 40 eV pass energy, step size of 0.1 eV) were collected on each of the surfaces. Survey and high‐resolution EDCs did not show any unexpected peaks, suggesting that surfaces were free from significant contamination from salts in the solution. High resolution EDCs were fitted with a 70 % Gaussian, 30 % Lorentzian line shape, except for Au 4f which used 10 % Gaussian, 90 % Lorentzian line shape. Linear backgrounds were used in the fitting of all high‐resolution EDCs, except for the Au 4f, where a Shirley‐type background was applied.

## Conflict of interest

The authors declare no conflict of interest.

## Supporting information

As a service to our authors and readers, this journal provides supporting information supplied by the authors. Such materials are peer reviewed and may be re‐organized for online delivery, but are not copy‐edited or typeset. Technical support issues arising from supporting information (other than missing files) should be addressed to the authors.

Supplementary
