# Supplementary material for: Elucidating the Influence of Electrical Potentials on the Formation of Charged Oligopeptide Self‐Assembled Monolayers on Gold
Source: Chemphyschem. 2021 Mar 5;22(7):684–92. doi: 10.1002/cphc.202000988 (PMC8048453; doi:10.1002/cphc.202000988)
Supplement: Supplementary file 1 — Supplementary [file CPHC-22-684-s001.pdf]

ChemPhysChem

Supporting Information

Elucidating the Influence of Electrical Potentials on the Formation of Charged Oligopeptide Self-Assembled Monolayers on Gold

Joshua S. Gibson and Paula M. Mendes*

Supporting Information

1. SPR response resulting from application of potential to surfaces in the presence of 1×PBS

As part of the experimental design in this study it was desirable to study the effect of an applied potential on the self-assembly of the charged oligopeptide, C5K, from a solution made up in 1×PBS. It was found that the application of a potential to the gold surface submerged in 1×PBS solution would cause a signal increase, as seen in Figure S1. For this reason it was decided that the potential would be applied 600 seconds prior to the injection of the thiols, to allow the signal changes from the electrical double layer to stabilise. It can be seen from Figure S1 that after 600 seconds (marked with a vertical dashed line) the SPR signals have stabilised for the potential ranges studied.

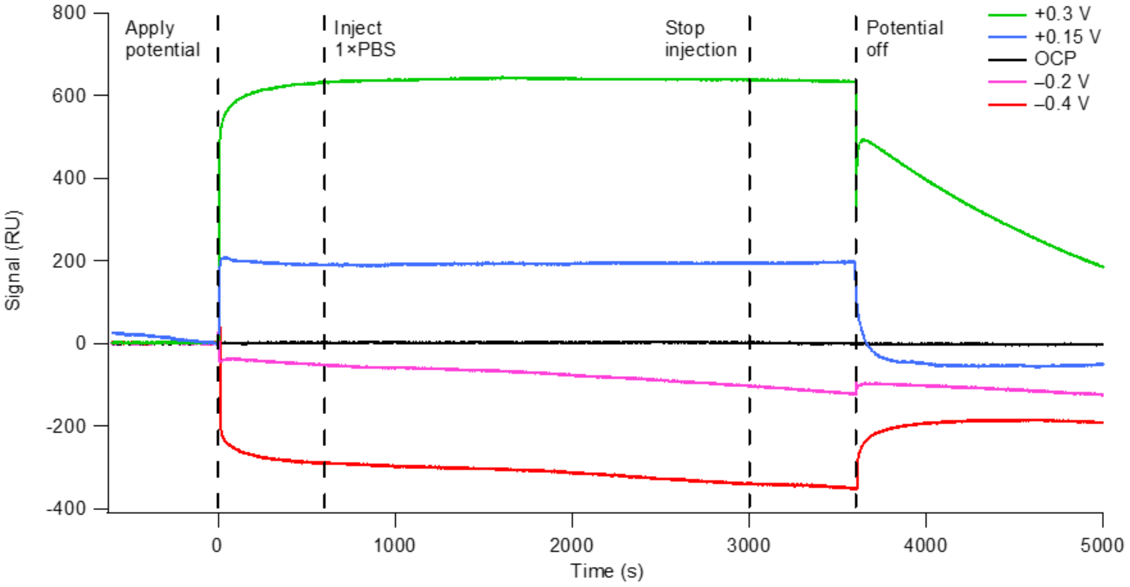

Figure S1. e-SPR signal response to the application of potentials between −0.4 V and +0.3 V to 1×PBS in the e-SPR cell. Injections of 1× PBS were performed to show that the injection system was clean (as there is no SPR response change after injection) and that the injection itself does not appear to change the near-surface structure.

2. Models used for fitting the XPS data of C5K on gold surfaces

Since only one monolayer of material is adsorbed the signal to noise ratios were quite low, meaning that for the C5K peaks a "linear" background was used, whilst for the gold peaks a "Shirley" background was used. To ensure a uniformity during fitting positional and FWHM constraints were used for the different chemical shifts, as shown in Table S1. Constrained peaks have been denoted, with constraints applied in relation to the "free" peaks. Area constraints were used only for the spin-orbit split components of the Au 4f spectra, where the area of the Au $4f_{5/2}$ peak is known to be 0.75 times that of the Au $4f_{7/2}$ component. The S 2p spectra have not been fitted with a doublet due to the very low signal-to-noise ratio meaning that it is not possible to resolve the closely spaced (ΔE = 1.1 eV) S $2p_{3/2}$ and S $2p_{1/2}$ spin-orbit split peaks, with a single S $2p_{total}$ peak being adequate to provide a peak area.

Table S1. Fitting constraints used for the C5K coated surfaces on gold substrates. Bold atoms indicate the environment that is being fitted or that the constraint is relative to.

Environment	Position (eV)	FWHM (eV)
C–**O**H (constrained)	C=**O** + 1.43	C=**O** × 1
C=**O** (free)	531.8 ± 0.1	—
**N**H_3^+ (constrained)	**N**H_2 + 1.85	**N**H_2 × 1
**N**H_2 (free)	400.0 ± 0.1	—
N–**C**=O (constrained)	**C**–C + 2.99	—
**C**–N (constrained)	**C**–C + 1.09	N–**C**=O × 1
**C**–C (free)	285.4 ± 0.2	—
**S** $2p_{total}$ (free)	162.3 ± 0.3	—
**Au** $4f_{5/2}$ (constrained)	Au $4f_{7/2}$ + 3.7	Au $4f_{7/2}$ × 1
**Au** $4f_{7/2}$ (free)	84.1 ± 0.1	—

3. Effect of C5K monolayer presence on adventitious hydrocarbon adsorption

The measured X-ray photoelectron spectra were also fitted in a way which applied constraints to the C 1s high-resolution spectra, such that the areas of the C 1s components for N−**C**=O, **C**−N and **C**−C were fixed to the stoichiometric ratios in the C5K molecule (with the ratio 6:11:16, or 1:1.83:2.67). Note that this is different to the way in which the data was fitted within the main manuscript, due to the reasonably low levels of adventitiously adsorbed material on the surfaces and the accepted presence of adventitiously adsorbed material on almost all XPS samples studied to date. For the fits including and adventitious C 1s component the FWHM were constrained in the same way as explained elsewhere in this manuscript. An additional peak, attributed to adventitious carbon, was allowed to be fitted to the high resolution C 1s region (Figure S2), with the constraints being that the peak position and FWHM of the adventitious carbon would be the same as that of the C−C environment in the C5K. The average area for the C_{Adven} component across all the C5K covered surfaces was 500 ± 1000 CPS, whilst for a bare gold surface the adventitious carbon component (red data, Figure S2b) had an area of 12100 CPS. As expected there was a large variation in the amount of adventitiously adsorbed carbon species across all samples and positions. This shows that the presence of the C5K overlayer appears to reduce the amount of carbon contamination by a factor of 10.

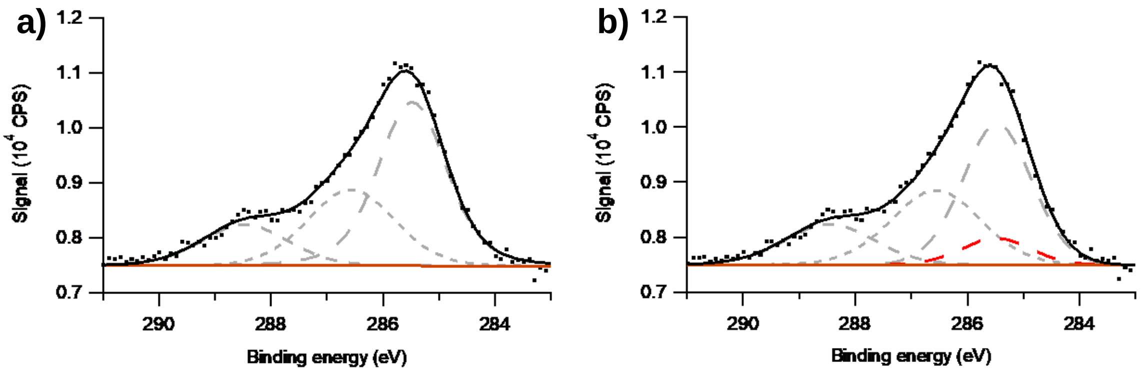

Figure S2. High resolution C 1s spectra for a C5K surface incubated at OCP showing the data fitted without (a) and (b) with an adventitious carbon peak (shown in red). When fitting with an adventitious carbon peak it is necessary to use constraints to fix the ratios of the other carbon environments in C5K (grey). The brown line represents the fitted linear background to the data with the black dots representing the experimental data and the black line showing the sum of all fitted peaks.

4. Bare gold surface before and after sputtering to determine I_0 for C5K coverage determination

Prior to forming a C5K surface, the gold chips were cleaned in piranha solution, rinsed in ultra-pure milli-Q water and dried in an argon stream and then immediately loaded into the electrochemical cell for incubation. It is expected that minimal contamination of the gold surface will occur during this time, due to the short timescales involved, and the well-known inertness of gold surfaces to most chemical species. After formation of the C5K surfaces they were rinsed in ultra-pure milli-Q water and stored in glass vials under an inert argon environment. Since the XPS analysis would not be performed immediately after formation of the C5K layers, a bare gold surface was also prepared. Bare gold was incubated in a 1×PBS solution for 90 minutes, with no applied potential (OCP). The bare gold chip served two purposes; to look at the amount of adventitious adsorption that occurred under these inert environments and to provide a bare gold baseline for the surface coverage determination. Figure S3 shows the high-resolution spectra for the Au 4f, C 1s and O 1s regions before and after sputtering with an Ar^+ ion gun (2 kV, 1μA emission, 3 mm × 3 mm raster), for 15 minutes. The area for the Au 4f spectrum for the cleaned surface was used in the surface coverage determination of the C5K on the gold substrates.

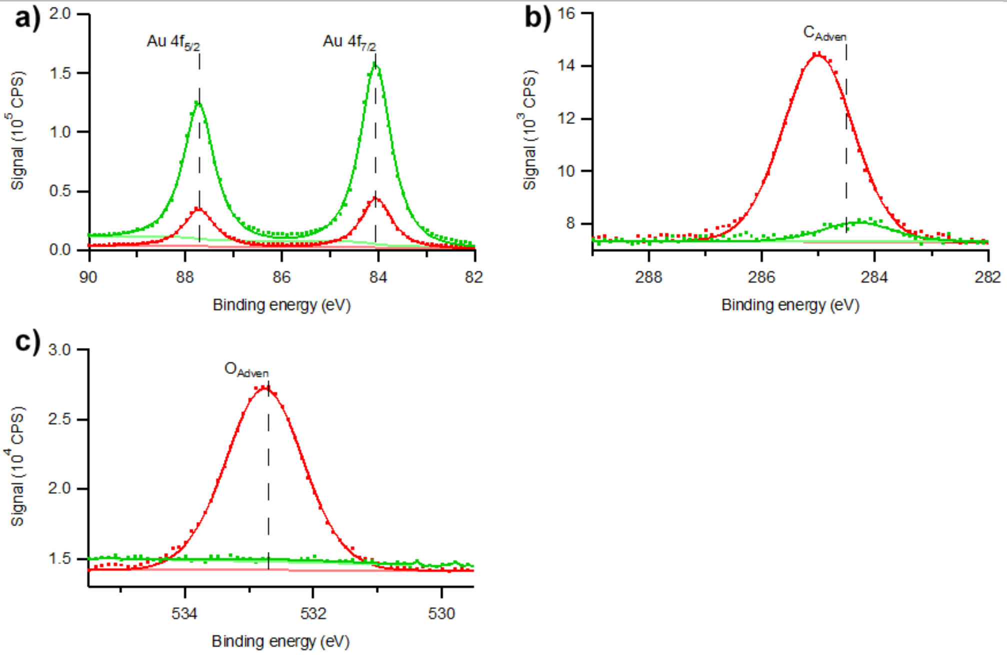

Figure S3. High resolution spectra for the (a) Au 4f, (b) C 1s and (c) O 1s regions for a gold surface that has been exposed to 1×PBS for 90 minutes as part of a null experiment. Data in red denotes the surface prior to Ar^+ ion sputtering, while the green data was collected from the surface after 15 minutes of Ar^+ ion sputtering. Collected data is shown with points, faint lines show the background while darker lines show the fitted components.
